# The malleable gut microbiome of juvenile rainbow trout (*Oncorhynchus mykiss*): Diet-dependent shifts of bacterial community structures

**DOI:** 10.1371/journal.pone.0177735

**Published:** 2017-05-12

**Authors:** Stéphanie Céline Michl, Jenni-Marie Ratten, Matt Beyer, Mario Hasler, Julie LaRoche, Carsten Schulz

**Affiliations:** 1 Gesellschaft für Marine Aquakultur mbH (GMA) Büsum, Büsum, Germany; 2 Department of Marine Aquaculture, Institute of Animal Breeding and Husbandry, Christian-Albrechts-Universität zu Kiel, Kiel, Germany; 3 GEOMAR Helmholtz Centre for Ocean Research Kiel, Kiel, Germany; 4 Department of Biology, Dalhousie University, Halifax, Canada; 5 Lehrfach Variationsstatistik, Christian-Albrechts-Universität zu Kiel, Kiel, Germany; Institut National de la Recherche Agronomique, FRANCE

## Abstract

Plant-derived protein sources are the most relevant substitutes for fishmeal in aquafeeds. Nevertheless, the effects of plant based diets on the intestinal microbiome especially of juvenile Rainbow trout (*Oncorhynchus mykiss*) are yet to be fully investigated. The present study demonstrates, based on 16S rDNA bacterial community profiling, that the intestinal microbiome of juvenile Rainbow trout is strongly affected by dietary plant protein inclusion levels. After first feeding of juveniles with either 0%, 50% or 97% of total dietary protein content derived from plants, statistically significant differences of the bacterial gut community for the three diet-types were detected, both at phylum and order level. The microbiome of juvenile fish consisted mainly of the phyla Proteobacteria, Firmicutes, Bacteroidetes, Fusobacteria and Actinobacteria, and thus fits the salmonid core microbiome suggested in previous studies. Dietary plant proteins significantly enhanced the relative abundance of the orders Lactobacillales, Bacillales and Pseudomonadales. Animal proteins in contrast significantly promoted Bacteroidales, Clostridiales, Vibrionales, Fusobacteriales and Alteromonadales. The overall alpha diversity significantly decreased with increasing plant protein inclusion levels and with age of experimental animals. In order to investigate permanent effects of the first feeding diet-type on the early development of the microbiome, a diet change was included in the study after 54 days, but no such effects could be detected. Instead, the microbiome of juvenile trout fry was highly dependent on the actual diet fed at the time of sampling.

## Introduction

With increasing knowledge about the impact of the gut microbiome on human health and nutritional status (see review by the Human Microbiome Project Consortium [1]), also an increase in studies about the fish microbiome can be observed, which is especially promoted by the availability of new technologies [2]. In recent years the development of rapid and precise culture-independent techniques like next-generation sequencing (NGS) has led to the discovery that enzyme producing bacteria within the fish intestinal microbiome can positively influence nutrient digestibility in several fish species by excreting digestive enzymes like amylase, cellulase, lipase or even phytase (see detailed review by Ray, Ghosh and Ringø [3]). For example, Fusobacteria and Bacteroidetes played an important role in the fermentation of plant material in Common carp (*Cyprinus carpio*) [4] and Firmicutes found in the gastro-intestinal (GI) tract of Grass carp (*Ctenopharyngodon idellus*) could utilize various polysaccharides, such as cellulose, xylan and hemicelluloses [5]. Several studies that were reviewed by Austin [6] furthermore reported that microorganisms in the fish gut are able to produce inhibitory compounds that can control the colonization of pathogens in the GI-tract. Lactic acid bacteria in Atlantic salmon (*Salmo salar*) for example, were shown to inhibit the intestinal colonization of the fish pathogen *Aeromonas salmonicida* [7] and plant-based diets seem to promote a protective effect against *Yersinia ruckeri* in juvenile Rainbow trout (*Oncorhynchus mykiss*) [8], possibly due to increased numbers of *Lactobacillaceae*.

Plant material as an alternative protein source is a crucial issue in modern aquaculture feed production. With significantly decreasing marine fish stocks and an increasing demand for fish as a protein source for human consumption, plant-derived proteins as an alternative feed source for fishmeal have been of high commercial and scientific interest during the past decades [9], especially for carnivorous species such as salmonids. However, although several studies demonstrated that juvenile and adult Rainbow trout can be successfully reared with plant material [10–14], the amount of fishmeal as protein source in salmonid fry feed is still greater than 50% [15]. This is probably due to the fact that very high inclusion levels of plant proteins in first feeding diets led to reduced growth accompanied by inflammation of the gastro-intestinal tract in most studies. Even though the importance of dietary effects on the gastro-intestinal microbiome of fish has been demonstrated in several publications, as reviewed by Ringø et al. [16], little is known on how early feeding of plant proteins is affecting the developing intestinal microbiome of juvenile carnivores. It has been shown for amberjack juveniles (*Seriola dumerili*) fed diets supplemented with soybean meal that the inclusion of heat-killed *Lactobacillus plantarum* has a positive impact on the utilization of dietary soybean meal [17], which implies interactions between the intestinal microbiota and the utilization of plant-derived protein sources. A recent study [18] demonstrated that first feeding initializes the colonization of the gut in Rainbow trout fry, but the subsequent development of the bacterial community structure is influenced by the diet-type. During the early development of vertebrates, specific nutritional stimuli can induce permanent effects on subsequent physiological processes; a concept that is known as nutritional programming and its consequences for human health have been approached in several studies as reviewed by Hanley et al. [19]. Nutritional programming has successfully been investigated with regard to carbohydrate metabolism in Rainbow trout [20, 21] and zebrafish [22, 23], as well as with regard to the utilization of plant-derived protein sources in trout [24, 25] and gilthead sea bream (*Sparus aurata*) [26]. The question arises whether the intestinal microbiome can also be programmed by the early feeding of plant-based diets. In zebrafish, intrinsic factors play an important role in the initial development of the microbial community structure [27], but whether these can outplay dietary modulations is unknown. The purpose of the current study was therefore to evaluate effects of first feeding diets with various levels of plant-derived proteins on the initial gut microbiome development of Rainbow trout fry and to examine whether or not a subsequent diet change during the juvenile stage would influence the diversity of the microbial community structure. It was hypothesized that due to the concept of nutritional programming the first feeding diet would promote an initial microbial community that is subsequently influencing diet-dependent alterations of the intestinal microbiome at later stages in life.

## Material & methods

### Experimental animals

The experiment was conducted at the “Gesellschaft für Marine Aquakultur mbH” (Büsum, Germany). Eyed Rainbow trout eggs (*Oncorhynchus mykiss*, Kamloops strain) were purchased from the trout farm “Forellenzucht Trostadt GbR” (Reurieth-Trostadt, Germany), originating from “Troutlodge Inc.” (Sumner, Washington USA). All animal handling procedures were approved by the animal welfare officer of the “Gesellschaft für Marine Aquakultur mbH” and the local authority of Schleswig-Holstein according to the German animal welfare law (TierSchG).

### Experimental diets

Three isonitrogenous and isoenergetic (on digestible matter; see Table 1) experimental diets (diet A, diet B and diet C) were pelletized in different particle sizes according to the needs of first feeding and growing fry. Diet A consisted exclusively of animal derived protein sources, contrasting diet C, which contained 97% of plant derived protein sources. Three percent of gelatin was needed for hardening during the manufacturing process of the first feeding diets. Diet B was intermediate between diet A and diet C with 50% plant and 50% animal protein sources. Amino acid composition of each diet was formulated according to the NRC [28] amino acid requirements for small Rainbow trout (0.2–20.0 g); likewise was the composition of the vitamin and mineral premixtures in accordance with NRC guidelines.

**Table 1:** Composition of experimental diets.

Ingredients (in % of dry matter)	Diet A	Diet B	Diet C
Fishmeal^1^	64.65	28.74	
Mussel meal^2^	2.13	2.00	
Blood meal^3^	6.14	0.96	
Shrimp meal^1^	8.65	6.00	
Corn gluten^4^		1.00	2.00
Soy protein concentrate^5^		5.00	5.00
Pea protein^6^		19.86	48.19
Rapeseed concentrate^7^		4.84	15.96
Wheat gluten^8^		10.00	2.79
Wheat starch^8^	6.43	3.38	2.00
Vitamin & Mineral Premixtures^9^	4.00	4.00	4.00
Linseed oil^10^	2.00	3.06	2.00
Fish oil^1^	3.00	4.78	9.07
Gelatine^11^	3.00	3.00	3.00
Bentonite^12^		3.38	5.97
Tryptophan^13^			0.03
**Proximate dietary composition (in % of dry matter)**			
Dry matter (in % of diet)	88.78	92.25	91.16
Crude protein	62.72	62.81	62.84
Crude fat	12.72	13.12	17.48
Crude ash	15.83	13.00	10.36
Gross energy (MJ kg^-1^)	21.37	22.15	23.42
Digestible energy (MJ kg^-1^)^14^	19.40	19.30	18.90
Amino acid composition* **(in % of diet)**			
Arginine	3.06	3.47	4.27
Histidine	1.32	1.18	1.27
Isoleucine	1.91	2.18	2.45
Leucine	3.91	4.03	4.49
Lysine	3.85	3.23	3.46
Methionine	1.30	1.05	0.78
Cystine	0.43	0.59	0.63
Phenylalanine	2.22	2.48	2.84
Tyrosine	1.01	1.48	1.58
Threonine	2.14	2.01	2.13
Valine	2.75	2.56	2.72
Alanine	3.36	2.74	2.57
Aspartic acid	4.93	4.94	5.99
Glutamic acid	6.57	9.66	9.92
Glycine	3.84	3.19	2.78
Proline	2.46	3.16	2.95
Serine	2.23	2.49	2.78
Fatty acid composition* **(in % of total fatty acids)**			
n-6 / n-3 *ratio*	0.28	0.64	0.97
Total n-6	6.59	15.60	19.81
Total n-3	23.86	24.20	20.32
ALA / LA *ratio*	1.78	0.97	0.63
Total C18:2n-6 (LA)	5.38	14.79	19.12
Total C18:3n-3 (ALA)	9.57	14.34	12.10
EPA / DHA *ratio*	0.76	0.88	1.04
Total C20:5n-3 (EPA)	4.90	3.72	3.21
Total C22:6n-3 (DHA)	6.47	4.22	3.10

^1^ Vereinigte Fischmehlwerke Cuxhaven GmbH & Co. KG, Cuxhaven, Germany

^2^ CRM—Coastal Research & Management, Kiel, Germany

^3^ SARVAL Ouest, Issé, France

^4^ Cargill Deutschland GmbH, Krefeld, Germany

^5^ EURODUNA Rohstoffe GmbH, Barmstedt, Germany

^6^ Emsland-Stärke GmbH, Emlichheim, Germany

^7^ Helm AG, Hamburg, Germany

^8^ Kröner Stärke GmbH, Ibbenbüren, Germany

^9^ Aller Aqua Mix 7188 Micro & 7180 Vit. STD., Golßen, Germany

^10^ Makana Produktion und Vertrieb GmbH, Offenbach, Germany

^11^ ARTI-Vital, Freyburg, Germany

^12^ DEL LAGO Bentonite, Castiglioni Pes y Cia, Buenos Aires, Argentina

^13^ Evonik Industries AG, Essen, Germany

^14^ Calculated—based on ADC values available from current literature

* Analysis was performed by Skretting ARC, Stavanger, Norway

### Experimental setup

**Fig 1:**
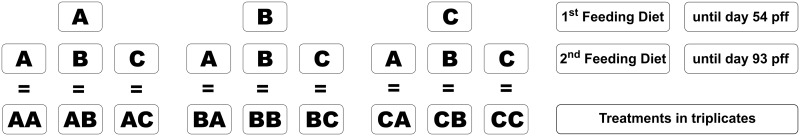
Scheme of the experimental design used in this study. The fishmeal diet A, the intermediate diet B and the plant-based diet C were fed as first feeding diet until day 54 post first feeding, which was the first sampling day for microbiome analysis. Afterwards fish of each dietary group were divided into three subgroups and the same three diets were fed as second feeding diet in a cross-over design until day 93 post first feeding, which was the second sampling day. The treatments reveal the nine resulting combinations of first and second feeding diet.

7500 eyed Rainbow trout eggs were randomly distributed among three commercial hatching troughs with 2500 fish in each trough that were integrated into a recirculating freshwater waterbody. Average temperature was 9.0 ± 0.2°C (data in mean ± SD) until hatching day and average oxygen concentration was 11.2 ± 0.3 mg l-1 from the day of arrival until hatching day. Hatching was induced by an increase in water temperature to 11.0°C and remained at 11.6 ± 1.1°C until the end of the experiment. Average oxygen concentration was 10.4 ± 0.5 mg l-1 and average pH was 7.6 throughout the whole feeding trial. First feeding was initiated on day 21 post hatch. Each hatching trough represented one of the three experimental groups for the first feeding period. Trout fry were fed with the first feeding diets A, B or C *ad libitum* with automatic feeders. Feeding frequency was once per hour for 19 days and subsequently reduced to a frequency of four times per day until 54 days post first feeding (pff). Dimmed light was provided from 06:00 am to 09:00 pm in order to make feed particles visible to the fish. On day 54 pff 1800 trout fry from each of the three hatching troughs were randomly distributed among nine 50 L aquaria integrated in the established recirculating system, resulting in a total of 27 aquaria. All diets were changed in a cross-over design (see Fig 1) and until day 93 pff each experimental group was fed four times per day with their second feeding diet—in total 3.8% of the total biomass. All second feeding diets were applied in triplicates. On days 54 and 93 pff, samples for microbiome analysis were taken and bodymass of experimental fish were determined.

### Sample preparation

In total, 150 fish were sampled for intestinal microbiome analysis. 15 fish were collected at the end of the first feeding period on day 54 pff (five animals from each of the three hatching troughs) and 135 animals at the end of the second feeding period on day 93 pff (five animals from each of the 27 aquaria, i.e. 15 fish from triplicate aquaria for each experimental treatment). Fish were fed two hours before sample collection and feed intake was visually monitored. Individual body weights of all collected fish were measured. Moreover, 20 additional fish per treatment were weighed at the end of the first feeding period and 60 additional fish per treatment at the end of the second feeding period (20 fish per triplicate aquarium). Prior to sampling, experimental animals were narcotized with MS222 (Tricaine methanesulfonate, E10521, Sigma-Aldrich Co. LLC.) and immediately killed by cutting the gill vein. The whole gastrointestinal tract was dissected on ice using sterile razor blades and with remaining gut content instantly frozen at -80°C.

### DNA extraction and purification

DNA extraction was performed with the Qiagen DNeasy^®^ Blood & Tissue DNA extraction kit according to the manufacturer’s protocol. First, gastro-intestinal samples (whole GI-tract, including remaining digesta) were thawed at 4°C and homogenised (KT Miccra D9 homogenizer) on ice for 30 seconds in 1 ml of a 5 mg ml^-1^ lysozyme (8259, Carl Roth) in TE-buffer solution (10 mM Tris-HCl, 1 mM EDTA). The homogenised solution was incubated for 30 min at 37°C. Next, the homogenate was gently vortexed and 80 μl were incubated for 60 min at 56°C in 200 μl of lysis buffer AL (provided in the extraction kit), 20 μl Proteinase K and 100 μl PBS (Solution without Ca-Mg, 733–2296, VWR). After incubation, 200 μl ethanol (96–100%) was added and further extraction steps were performed according to the manufacturer’s protocol for purification of total DNA from animal tissue. According to recommendations by Qiagen, two extra washing steps with the provided buffers AW1 and AW2, as well as an extra centrifugation step of 1 min at maximum speed before elution were included in the procedure. DNA purification was initiated with RNase A (Qiagen) digestion (1 mg ml^-1^ in DEPC water), followed by inactivation remaining microbial DNases at 70°C for 15 min. This working solution was added to each sample to obtain a final concentration of 100 μg ml^-1^ RNase A and incubated for 30 min at 60°C. This was followed by a DNA clean-up step using the NucleoSpin^®^ gDNA clean-up kit (Machery-Nagel) following the manufacturer’s protocol including all recommended steps.

### 16S rDNA PCR amplification

All DNA samples were amplified by PCR targeting the 16S rRNA gene sequence (regions V6-V8). The final PCR reaction volume was 20 μl including 4 μl CG buffer, 0.6 μl DMSO, 0.4 μl dNTP (10 mM), 0.4 μl of each primer, 0.2 μl Phusion high-fidelity polymerase (Thermo Fisher Scientific Inc.), 12 μl DEPC H_2_O and 2 μl of DNA template. The final primer concentration was 0.5 μM. Primers used were B969F (5’-ACG CGH NRA ACC TTA CC-3’) and BA1406R (5’-ACG GGC RGT GWG TRC AA-3’) from IDT (Integrated DNA Technologies, Inc.) according to [29]. Cycling protocol was as follows: 98°C for 3 min, 35 cycles of 98°C for 10 sec., 54°C for 30 sec. and a final extension at 72°C for 1 min., and finally 72°C for 10 min Results of the PCR were verified on a 1.1% agarose gel. DNA samples were stained with SYBR safe DNA gel stain (Invitrogen^™^, Thermo Fisher Scientific Inc.) and images were analysed using a gel imaging box (G:BOX, Syngene). Afterwards, samples were gel extracted using the QIAquick Gel Extraction Kit (Qiagen) following the manufacturer’s protocol and DNA concentrations were determined using a NanoDrop 2000 UV-Vis Spectrophotometer (Thermo Fisher Scientific Inc.). Amplified products were cleaned and normalized using the SequalPrep Normalization Plate Kit (Invitrogen^™^, Thermo Fisher Scientific Inc.). Subsequently, the samples were multiplexed at equal volumes, quantified with Qubit (Invitrogen^™^, Thermo Fisher Scientific Inc.) and loaded into the Illumina MiSeq platform as a 20 pM final denatured library according to manufacturer’s specifications.

### Sequencing and bioinformatics workflow

All raw sequences used in this study are stored at the Sequence Read Archive (SRA) and can be accessed via the SRA accession number SRP090805 or the BioProject ID PRJNA344407. The amplified 16S rDNA fragments were sequenced at the Integrated Microbiome Resource lab (IMR) at Dalhousie University (Halifax, Canada) using an Illumina MiSeq platform and following the procedure described in [30]. Raw sequences were analysed with QIIME (Quantitative Insight Into Microbial Ecology) for the analysis of high-throughput community sequencing data [31] using the 16S amplicon analysis procedure of IMR [32]. The following quality control methods were applied: forward and reverse reads were matched using PEAR (Paired-end rEAd merger; [33]), sequences with unidentified nucleotides, with mitochondrial and chloroplast DNA sequences [34], and chimeric DNA molecules were removed (using UCHIME; [35]). Open-reference OTU (Operational Taxonomic Units) picking was performed using the picking methods *sortmerna* and *sumaclust* [36]. Reads were clustered against the reference database Greengene [37] and OTUs were grouped together based on 97% sequence similarity. Low confidence (i.e. false-positive) OTUs were subsequently removed. The threshold for removing low confidence reads was set to 0.1%, which has been reported by Illumina to be the maximum of bleed through reads on the Illumina MiSeq platform. The collection of sequences was rarified to 1000 reads per sample, which was a compromise between what has been suggested for gut samples by Hamady & Knight [38] and a substantial amount of samples from each tank remaining for statistical analysis.

### Statistical analysis

The statistical software R [39] and QIIME were used to evaluate the data, generated on days 54 and 93 post first feeding. Important to note here is the small number of samples obtained for diet C at sampling day 54 post first feeding: from five GI-tract samples extracted, only three samples showed substantial sequencing results and could thus be integrated into the statistical analysis. Based on the complete OTU table generated during the QIIME workflow the number of observed distinct OTUs, the good’s coverage estimator, the Chao1 richness estimator, the Simpson’s evenness measure E and the Shannon diversity index H’ were calculated. Differences between bacterial communities in relation to the dietary treatment or sampling day were visualised by nonmetric multidimensional scaling (NMDS) based on a Bray-Curtis dissimilarity matrix of Hellinger-transformed abundance data on order level using the R package vegan [40]. A stress factor was calculated to provide a way of determining how well original data is represented in the ordination space. The core microbiota of fish fed the different experimental diets at the end of the first feeding period (dpff 54) and at the end of the second feeding period (dpff 93) was calculated with QIIME by using the complete OTU table generated during bioinformatics workflow. The core microbiota of the present study was defined as OTUs present in 80% of samples from each dietary treatment. By using an open access web tool, Venn diagrams were drawn to visualize the core microbiota (http://bioinformatics.psb.ugent.be/webtools/Venn/). The core microbiota on day 93 pff was calculated for the diets fed during the second feeding period only, irrespective of the first feeding diet. Those samples were pooled for their first feeding diets, since there was no significant impact of the first feeding diet on the microbial community at the end of the second feeding period.

Further statistical analysis of the data was performed as follows. For an extended version of the statistical analysis, please see S1 Text.

The dietary influence on alpha diversity and on the top-five most abundant phyla was evaluated by appropriate statistical models [41–43], analyses of variances (ANOVA) and subsequent multiple contrast tests [44–46]. A significant interaction of the first and the second feeding diet was generally considered as nutritional programming effect of the first feeding diet. Statistical differences between the two sampling points (day 54 pff and day 93 pff) were evaluated for continuously fed fish by using the same procedure.

Furthermore, the dietary influence on the bacterial community structure was tested via Principal Component Analysis (PCA) [47] by using Hellinger-transformed bacterial abundance data on order level. The principal components (PC) with greatest influence on overall variability were selected via Broken-Stick-Criterion [48]. An appropriate multivariate model was established based on those PC’s, followed by ANOVA and multiple contrast tests [49, 50]. Statistical differences between the two sampling points (day 54 pff and day 93 pff) were evaluated for continuously fed fish as previously described.

The PC’s with the highest contribution to the overall variation were further examined for the individual contribution of specific bacterial orders to the cumulative variance. The top-ten orders with the highest contribution to the cumulative variance were selected and multiple contrast tests were performed as described before [49, 50], respectively for each PC. Thus, specific bacterial orders could be identified that were significantly promoted by a certain diet-type.

Finally, a correlation analysis based on Spearman ranks was conducted in order to evaluate a possible relation between the bodymass of individual fish and the first two principal components of the foregoing PCA. The correlation analysis was repeated for each of the second feeding diets. For PC2 a significant correlation was found and the top ten orders with the highest loadings on this PC were used again in a Spearman ranks correlation analysis to test possible relations of a specific bacterial order to bodymass.

## Results

### Growth performance

**Fig 2:**
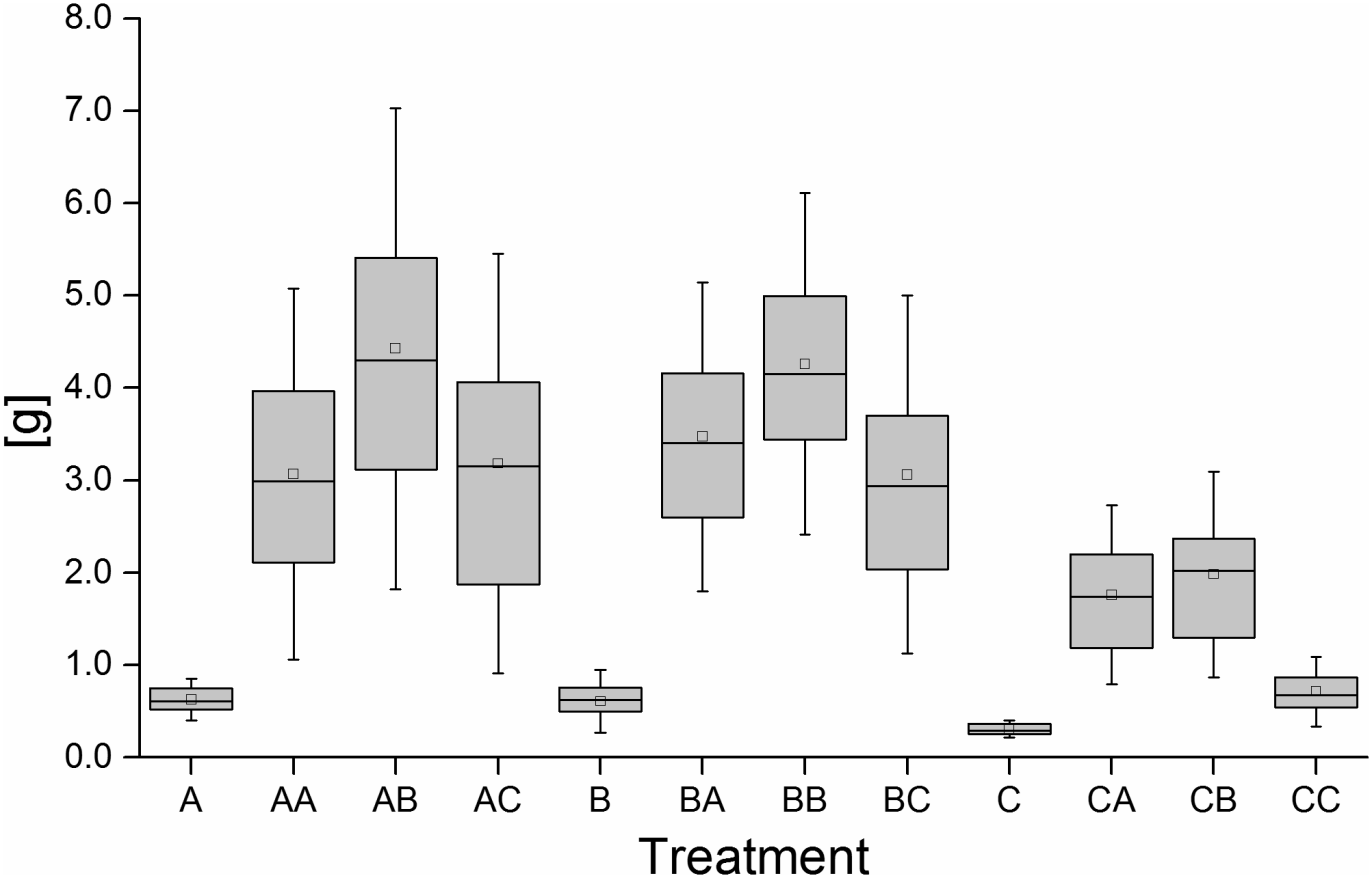
Final bodymass of experimental animals in relation to the corresponding treatment. Data is presented as boxplots with median, 25- and 75-percentiles and standard deviation as whiskers. Mean bodymass is indicated by open rectangles. Sample size is 25 individuals per treatment at the end of the first feeding period and 75 individuals at the end of the second feeding period (25 fish per tank, 3 tanks per treatment).

Fig 2 shows the final bodymass of fish at the end of the first and the second feeding period. The body mass of fish continuously fed with diet C is significantly reduced in comparison to the other treatments. Interestingly, the second feeding diet B also promoted better growth than the second feeding diet A.

### Alpha diversity of the GI tract microbiome

**Fig 3:**
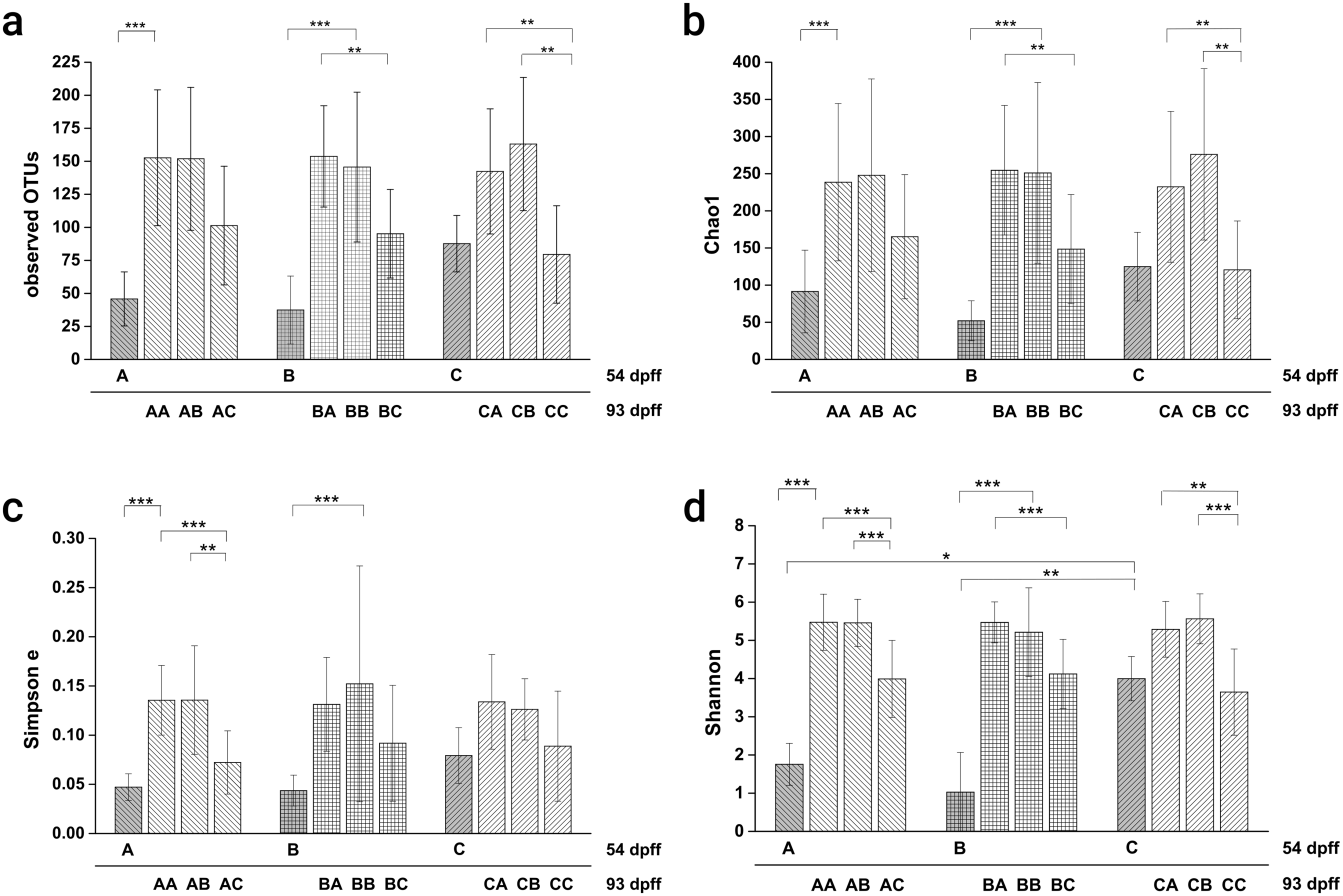
Alpha diversity indices in relation to the dietary treatment and sampling day. The number of observed OTUs (Fig 3a), Chao1 richness estimator (Fig 3b), Simpson’s evenness measure (Fig 3c) and Shannon diversity index (Fig 3d) are presented. Statistically significant differences between treatments or between sampling days for continuously fed diets are indicated with asterisks: p<0.05 (*), p<0.01 (**), p<0.001 (***).

Alpha diversity indices were calculated for data of both sampling days (see Fig 3). At the end of the first feeding period, only the Shannon diversity index differed significantly between the three diets. The index was significantly higher (p<0.05) for fish fed diet C than for fish fed diet A or diet B. All calculated indices increased significantly from day 54 to day 93 pff for individuals from treatments AA and BB. In contrast, diversity indices remained constant between the two sampling points for fish from treatment CC. Neither a significant effect of the first feeding diet on any of the calculated indices, nor a statistical interaction between the first and the second feeding diet was found; however, the second feeding diet was significantly influencing all alpha diversity indices. Multiple contrast tests revealed a significant decrease of all indices in fish of the second feeding diet C compared to the second feeding diets A and B. Good’s coverage estimators were 0.97 ± 0.01, 0.98 ± 0.01 and 0.97 ± 0.02 (diet A, diet B and diet C, respectively) for samples obtained on dpff 54. For samples obtained on dpff 93, good’s coverage estimators were 0.93 ± 0.03, 0.93 ± 0.04 and 0.95 ± 0.03 (treatments AA, AB and AC), 0.93 ± 0.03, 0.93 ± 0.03 and 0.96 ± 0.02 (treatments BA, BB and BC), and 0.94 ± 0.03, 0.92 ± 0.03 and 0.97 ± 0.02 (treatments CA, CB and CC).

### Relative abundance of bacterial phyla

**Fig 4:**
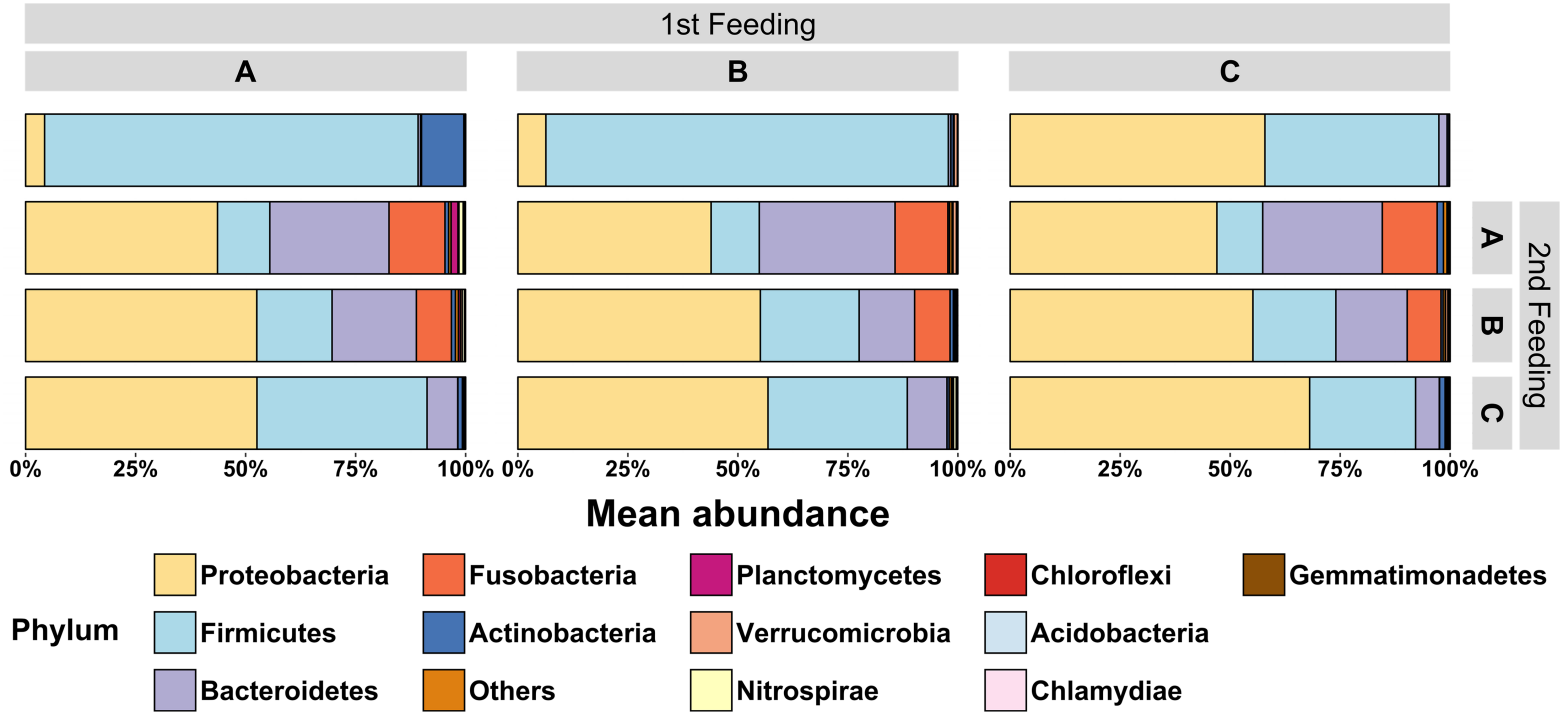
Mean relative abundance of phyla present in the intestinal tract samples of trout fry in relation to the dietary treatment. The top 13 phyla present in at least 10% of all samples and counting for at least 1% of all observed OTUs were included in the analysis; the remaining phyla were aggregated into “Others”.

Fig 4 presents the relative mean abundance of all phyla accounting for at least 1% of all observed OTUs and present in at least 10% of all samples. The top five most abundant phyla were Proteobacteria, Firmicutes, Bacteroidetes, Fusobacteria and Actinobacteria (see Table 2). On average they represented 98% of all sequences obtained. At the end of the first feeding period the relative abundance of individual phyla differed: diet C promoted significantly higher abundances of Proteobacteria (p<0.05) in comparison to diet B and diet A, in which Firmicutes dominated instead (p<0.05). Abundances of Actinobacteria were significantly increased (p<0.05) in fish of diet group A compared to fish of the diet groups B and C. The relative abundances of the phyla Bacteroidetes and Fusobacteria however did not differ between diets at the end of the first feeding period.

**Table 2:** Mean relative abundance in percent of the top five phyla found in GI tract samples of fish for each treatment at two different sampling days.

	Mean abundance of phyla in [%]					
Treatment	dpff	Proteobacteria	Firmicutes	Bacteroidetes	Fusobacteria	Actinobacteria
A	54	4.3 ± 5.6^a,^*	84.9 ± 4.2^a,^*	0.6 ± 0.4*	0.2 ± 0.3*	9.6 ± 4.3^a,^*
AA	93	43.6 ± 8.8*	11.9 ± 8.1*^,A^	27.1 ± 11.0*^,A^	12.7 ± 7.9*^,A^	0.8 ±1.3*
AB	93	52.5 ± 9.6	17.1 ± 6.6^A^	19.2 ± 10.6^A,B^	8.0 ± 4.3^A^	0.9 ± 2.0
AC	93	52.6 ± 19.2	38.7 ± 23.4^B^	7.0 ± 12.7^B^	0.0^B^	1.0 ± 2.5
B	54	6.4 ± 9.1^a,^*	91.5 ± 11.9^a,^*	0.6 ± 0.8*	0.0*	0.6 ± 0.6^b^
BA	93	43.9 ± 13.9	10.9 ± 6.2^A^	30.9 ± 12.5^A^	12.0 ± 6.7^A^	0.4 ± 0.3
BB	93	55.1 ± 16.8*	22.4 ± 22.6*^,A,B^	12.6 ± 4.6*^,B^	8.1 ± 4.8*^,A^	0.8 ± 0.9
BC	93	56.8 ± 12.1	31.7 ± 14.0^B^	9.0 ± 11.5^B^	0.0^B^	0.5 ± 0.9
C	54	57.9 ± 11.9^b^	39.5 ± 12.4^b^	1.9 ± 1.2	0.0	0.4 ± 0.1^b^
CA	93	46.9 ± 11.3	10.4 ± 5.2	27.2 ± 9.0^A^	12.5 ± 5.9^A^	1.4 ± 3.4
CB	93	55.2 ± 11.7	18.8 ± 16.2	16.2 ± 13.5^A,B^	7.7 ± 5.2^A^	0.5 ± 0.4
CC	93	68.0 ± 20.2	24.1 ± 20.1	5.4 ± 8.0^B^	0.0^B^	1.3 ± 2.2

Statistically significant differences between the three first feeding diets A, B and C are indicated with different lower case letters. Statistically significant differences between treatments after the second feeding period are indicated with different upper case letters, separate for the respective first feeding diet. Statistically significant differences between sampling days for continuously fed diets are indicated with asterisks.

After the diet change, no significant interaction between the first feeding diet and the second feeding diet was detected for the top five phyla. Instead, the mean abundance of several phyla was again significantly influenced by the second feeding diet. In individuals of the treatments AA and BB, Proteobacteria, Bacteroidetes and Fusobacteria significantly (p<0.001) increased in relative abundance from day 54 pff to day 93 pff. Firmicutes in contrast significantly decreased in both treatments, but Actinobacteria decreased only for fish of treatment AA. No differences were found between sampling days for fish of treatment CC. The relative abundance of Proteobacteria did not differ significantly between most of the second feeding diets, even though Proteobacteria were generally less abundant in fish fed with diet A as second feeding diet. Similar effects could be observed for the phylum Firmicutes: the relative abundance significantly increased (p<0.05) when diet C was used as second feeding diet in contrast to diet A and diet B. Bacteroidetes in contrast were significantly more abundant (p<0.05) when fish were fed with diet A as second feeding diet. Alike Bacteroidetes, relative abundances of Fusobacteria were significantly higher (p<0.01) when diet B or diet A were fed as second feeding diet. Abundances of Actinobacteria however were low and not significantly different for any of the treatments.

### The influence of diets and bodymass on the bacterial community

**Fig 5:**
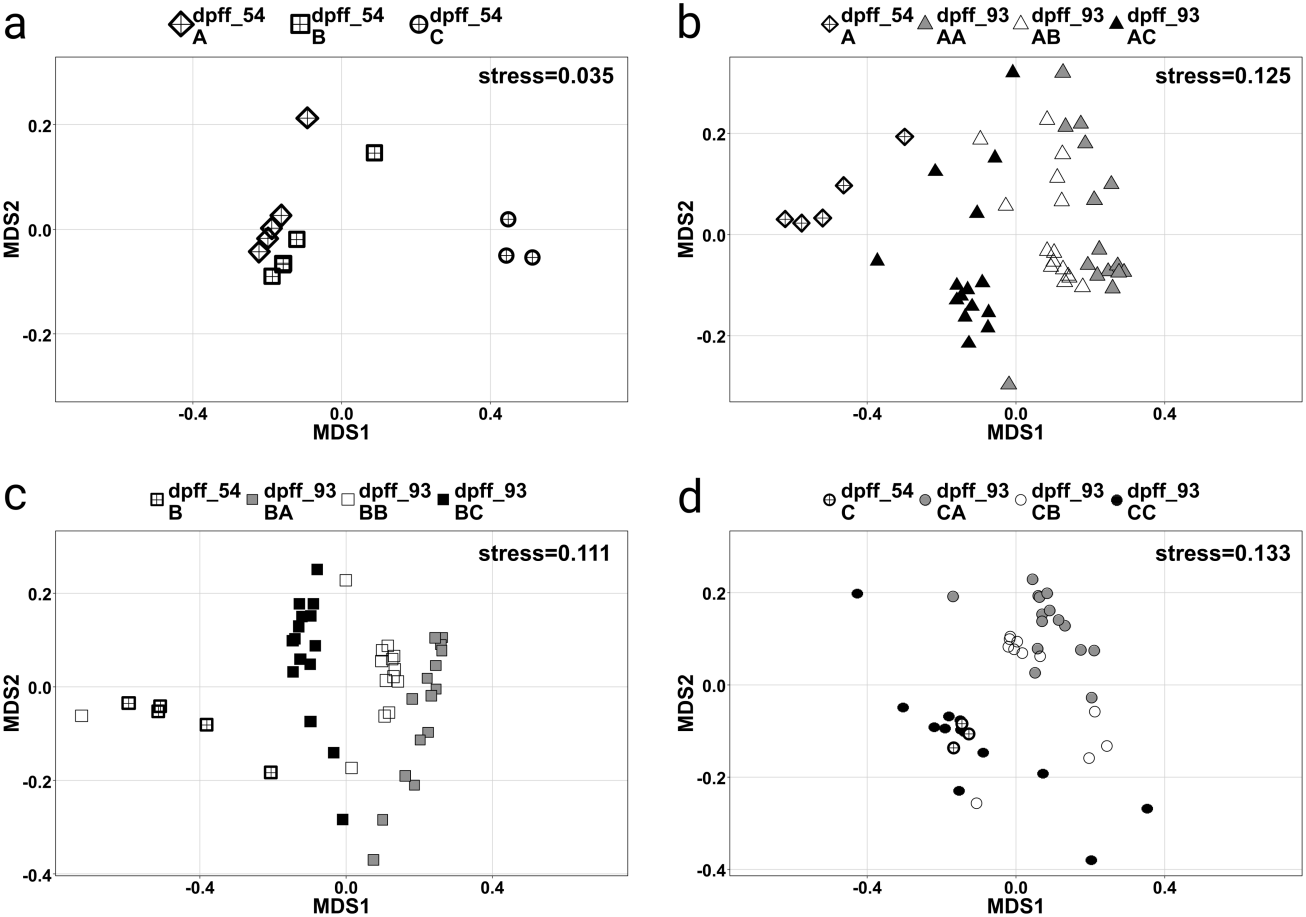
Nonmetric multidimensional scaling (NMDS) plots of the microbiomes of individual intestinal samples in relation to treatment and sampling day. MDS1 and MDS2 represent the two axes of the two-dimensional ordination space. Each point represents the microbiome of one individual fish. The stress-level shown in each plot indicates how well the individual distances between objects are represented (between 0 and 1; the closer to 0, the better are original data points represented in the ordination space). Plot (a) shows a comparison of samples by first feeding diet on day 54 pff. Plots (b), (c) and (d) compare the samples by second feeding diet on day 93 pff and continuously fed fish for both sampling days. The proximity between points represents the similarity of their microbiomes.

Four NMDS-plots are presented in Fig 5. Fig 5a shows that the analysis resulted in distinct grouping of fish from the same experimental groups. Additionally, the gut microbiome of fish fed diet A is very similar to the microbiome of fish fed diet B. In contrast, the data points of fish fed diet C are significantly offset, indicating a microbiome composition that is different from diet A and B, as confirmed by a statistical model (p<0.01).

Fig 5a–5d illustrate the microbial communities at the end of the first feeding period in relation to their corresponding communities at the end of the second feeding period. The stress level is less than 0.2 for all three plots and microbiomes from the same treatment clearly cluster together. The microbiomes of fish fed the second feeding diets A and B are more similar than to those of the second feeding diet C—independent of the first feeding diet. Furthermore, fish of the treatments AA and BB are clearly separated by the two sampling days which was further verified by the statistical model. Interestingly, fish of treatment CC had a similar gut microbiome on both sampling days.

The Principal Component Analysis revealed that PC1 already explained 41% and PC2 22% of the total variance observed in the dataset obtained at the end of the second feeding period. The first six principal components explained together 84% of the variability. No interaction between the first and the second feeding diet could be observed and only the second feeding diet significantly influenced (p<0.001) the microbial community composition. Furthermore, significant (p<0.001) differences of the gut microbiome for the three second feeding diets were found. The following orders were identified to be strongly influenced (p<0.001) by the second feeding diets A and B: Bacteroidales, Clostridiales, Lactobacillales, Bacillales and Pseudomonadales. Three additional orders were significantly different between diet A and diet C: Vibrionales, Fusobacteriales and Alteromonadales, and except for Lactobacillales, the same orders were found significantly altered in their abundance between diet B and diet C.

**Fig 6:**
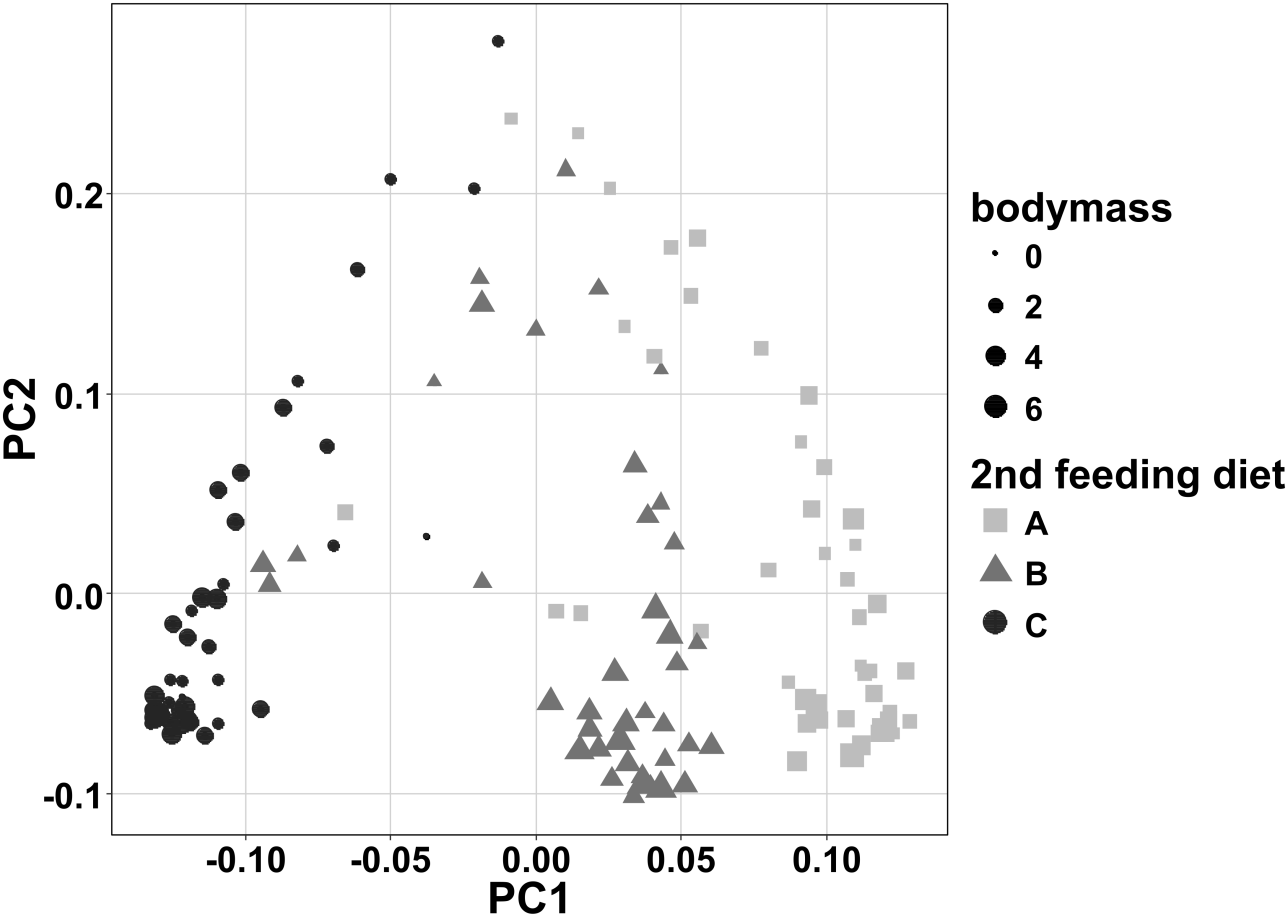
Graphical visualisation of the Principal Component Analysis (PCA) in relation to the second feeding diet and the individual bodymass. The first two principal components PC1 and PC2 (together representing 63% of the variance explained) are presented as axes of the ordination space. Each point represents the intestinal microbiome of one individual fish. Data were pooled for the first feeding diet according to the results of the multivariate model. Different gray shades and shapes of points indicate the three second feeding diets A, B and C. The size of the points indicates the final bodymass of each fish categorized as 0 (i.e. between 0.0g and 2.0g), 2 (i.e. between 2.1g and 4.0g), 4 (i.e. between 2.1g and 4.0g) and 6 (i.e. >6.0g).

The PCA plot (Fig 6) additionally indicates that the final bodymass plays a role for the data separation along the axis of PC2. The correlation analysis revealed a strong correlation (ρ = -0.476; p = 1.4e^-8^) between PC2 and the bodymass of individual fish. This correlation was persistent when dietary subgroups were tested. For all three second feeding diets PC2 remained significantly correlated to bodymass (diet A and B: p<0.001, diet C: p<0.05). From the ten orders with the highest loadings on PC2, especially Lactobacillales was highly correlated (ρ = 0.400; p = 2.9e^-6^) to bodymass.

### The core microbiome of experimental groups

**Fig 7:**
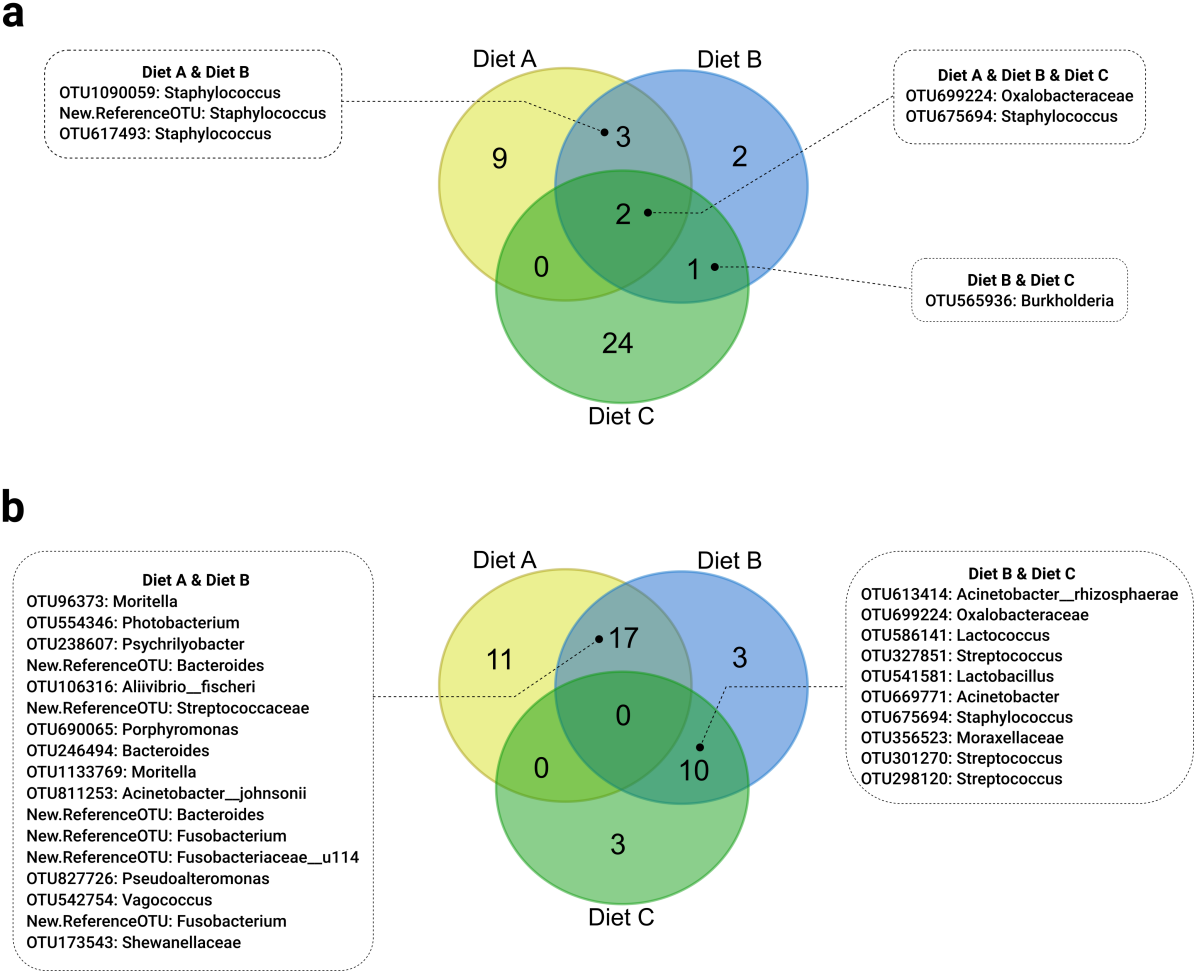
Venn diagram presenting the shared OTUs of fish fed the experimental diets. Presented are the numbers of OTUs present in at least 80% of all samples from one of the experimental groups fed one of the three experimental diets A, B or C. The numbers in the overlapping circles indicate OTUs shared by either two or three experimental groups. Plot (a) visualizes the core microbiota at the end of the first feeding period (54 dpff). Plot (b) visualizes the core microbiota at the end of the second feeding period (93 dpff). All samples were pooled for their respective first feeding diet and analyzed only for the second feeding diet.

At the end of the first feeding period (54 dpff) only two OTUs were detected as core microbiome in fish of all three first feeding diets: one at genus-level, *Staphylococcus* and one at family-level, Oxalobacteraceae (Fig 7a). No OTUs were shared by fish fed diet A or diet C. Three different OTUs belonging to the genus *Staphylococcus* were shared only by fish fed diet A or diet B; and one OTU of the genus *Burkholderia* was shared by fish fed diet B or diet C.

At the end of the second feeding period (93 dpff) all fish were pooled for their first feeding diet because of the non-significant interaction of first and second feeding diets. There were no OTUs shared by fish fed any of the three second feeding diets (Fig 7b). A core microbiome of 17 OTUs was found for fish fed diet A or B. Two OTUs on species level: *Aliivibrio fischeri* and *Acinetobacter johnsonii*; 13 OTUs belonging to eight different genera: *Bacteroides*, *Fusobacterium*, *Moritella*, *Photobacterium*, *Porphyromonas*, *Pseudoalteromonas*, *Psychrilyobacter*, *Vagococcus;* three OTUs on family level: Fusobacteriaceae, Shewanellaceae, Streptococcaceae. For fish fed diet B or C a core microbiome of ten OTUs could be identified. One OTU on species level: *Acinetobacter rhizosphaerae*; seven OTUs belonging to five diefferent genera: *Acinetobacter*, *Lactobacillus*, *Lactococcus*, *Staphylococcus*, *Streptococcus*; two OTUs on family level: Moraxellaceae and Oxalobacteraceae. Please refer to S1 Table for a complete list of the core microbiota.

## Discussion

### The influence of diets and bodymass on the microbiome

In this study different dietary plant protein inclusion levels were hypothesized to affect the intestinal environment of fish and thus alter the conditions for bacterial communities present in the gastro-intestinal tract. The intestinal microbiome of fish can be substantially influenced by several factors from the external and the internal environment, such as diet [51–54], feed intake [55], surrounding habitat [56] and genetic background [57]. Nutritional programming effects of the first feeding diet on the intestinal microbiome and the influence of a subsequent diet change have also been evaluated in the current study. However, the results of the analysis revealed that the microbial community is significantly determined by the diet fed at the point of sampling and that the first feeding diet is not programming the intestinal microbiome of trout fry. The present study clearly demonstrated that the microbiome can be manipulated during several life stages of Rainbow trout, which has already been suggested [18] and what is well-known from human studies. It has been demonstrated [58] that even a short-term consumption of a new diet alters the human gut microbiome rapidly. Order-specific analysis conducted in the current study revealed that Lactobacillales, Bacillales and Pseudomonadales were specifically promoted when plant proteins were included in the diet. In contrast, Bacteroidales, Clostridiales, Vibrionales, Fusobacteriales and Alteromonadales were significantly promoted when animal proteins were included in the diet. Lactobacillales, for example, produce lactic acid as a final metabolic product from the fermentation of carbohydrates and thus it is not surprising that plant meals promote an increase of this order. Furthermore, *Lactococcus* has been identified as predominantly genus of Lactic Acid Bacteria (LAB) in Rainbow trout, significantly inhibiting several fish pathogens [59], which could be beneficial during the early development of first feeding trout. Also in Atlantic salmon, LAB have been demonstrated to act anti-pathogenic [60]. Bacteroidales on the other hand include several microorganisms that are bile-resistant and related to protein fermentation. The differences between diet B and diet A were generally smaller than between diet C and diet A. Accordingly, the PCA plot revealed that the microbiomes of fish cluster by the second feeding diet and all NMDS plots indicated that the distances between objects reflect the inclusion level of plant proteins: individuals fed the mixed diet B were placed in-between both extreme diets A and C, with shorter distances towards the animal protein based diet and with longer distances towards the plant protein based diet. The final bodymass also significantly correlated with the intestinal microbial community at the end of the second feeding period. It can only be speculated what influencing factors cause this correlation. One possibility could be stress: Even though the stocking density in each tank was according to approved recommendations for small trout fry [61], high variation in growth rates between individual fish could have caused aggression, territorial or dominant behavior and thus lead to a higher stress level in smaller fish. It has been demonstrated that stress can induce a change in the microbiota of brook charr (*Salvelinus fontinalis*) [62] and thus, stress could be a reason for secondary bodymass dependent microbial community differences as observed in the present experiment. It was not possible to monitor this problem quantitatively during the experiment, but obviously big fish controlling the rest of the group (i.e. preventing from feeding or aggressive biting) were removed from the experimental group.

### Relative abundance of phyla

The development of diet dependent OTUs can also be observed in the analysis of relative abundances of phyla. As previously stated, Firmicutes played a significant role in the early intestinal microbiome of trout fry after the first feeding period. The abundance of Firmicutes was significantly higher when animal protein was used in the first feeding diets. In second feeding diets however, the abundance of Firmicutes was strongly reduced. These findings appear to be contrary to previous studies: Ingerslev et al. [18] for instance found Proteobacteria being significantly more abundant in early trout fry that have been fed with a fishmeal based diet until 49 days post first feeding. Nevertheless, their study also revealed an ontogenetic influence on the abundance of phyla, since 26 days post first feeding Bacteroidetes represented the group of most abundant OTUs. Another study evaluating the influence of plant proteins on the intestinal microbiome of larger Rainbow trout found Proteobacteria and Firmicutes to be the most abundant bacterial phylum present in both, fishmeal and plant-protein based diets [51], although with variations of OTUs between diet-types. Animal proteins used in the current study promoted a significant increase of Proteobacteria, Bacteroidetes and Fusobacteria during the second feeding period until day 93 post first feeding and displaced the high abundance of Firmicutes. Tenericutes had been found being the most abundant phylum in Rainbow trout fed a commercial diet [63], but the study already stated that for example the applied method (DGGE vs. 16S rDNA vs. cpn60 gene analysis, or the amplified regions V1-V3 vs. V5 vs. V6-V8) could have an effect on the differences in gut bacterial communities. Furthermore, the diets used in the current study not only contained fishmeal as animal protein source, but also shrimp meal, mussel meal and blood meal—together 26% of the amount of fishmeal. Since the focus of this study was not to evaluate individual protein sources, but the nutritional programming effect of first feeding diets, specific differences to other studies could also result from the used protein sources. The observed phyla in general were however quite consistent with previous results found for salmonids [51, 56, 64]. The core microbiome analysis performed in the present study demonstrated that fish fed either a fishmeal or a plant-protein based diet only share two OTUs after the first feeding period and none at the end of the second feeding period. This is contrasting previous results reporting that Rainbow trout has a rather resistant microbiome towards dietary variations [64]. This might be true on higher taxonomic levels, but the very low level of shared OTUs on genus or species level as observed in the current experiment might indicate that strong dietary effects can be rather observed on lower taxonomic levels. When fishmeal-free diets were applied to Atlantic salmon, for example, strong dietary effects on the relative abundance of *Lactobacillales* could be observed [54]. The microbiome of Atlantic salmon has just recently been shown to exhibit distinct differences between the intestinal compartments, and also between the digesta and mucosa [65]. It has been furthermore reported that dietary modulation effects on the intestinal microbiome of Atlantic salmon are different between intestinal compartments [52]. Those results suggest that future studies about dietary impacts on the intestinal microbiome should distinguish between effects on the allochthonous and autochthonous bacterial community, as well as between effects on the different intestinal compartments—which is, however, difficult to address in small fry.

### Alpha diversity values

Our results also demonstrate that not only the bacterial community structure in general is strongly affected by the level of plant proteins used, but also the species diversity of this community. The number of observed OTUs was statistically not influenced by the type of diet at the end of the first feeding period, which might however be due to the reduced sample size for diet C. The diet change and the following second feeding period however generated significant differences between treatments. At the end of the second feeding period (93 dpff) the number of observed OTUs significantly increased when fish were continuously fed with diet A or diet B. It can be speculated whether the stable number of OTUs present in samples of fish continuously fed with diet C is related to the significantly reduced growth or to the already higher number of observed OTUs after the first feeding period. Nevertheless, species richness is increasing after the diet change—independent of the first feeding diet. Species richness can only be affected by three processes [66]: speciation, extinction and dispersal. An increase in species richness could therefore be the result of colonization by rare (i.e. relatively less abundant) bacterial species or taxa. Within the second feeding period, new bacteria colonized the GI tract of all fish; the amount however was determined by the inclusion level of plant proteins. A significant increase of bacterial 16S rDNA has also been observed in Rainbow trout fry [18] during the first four weeks after first feeding and so did Shannon diversity, although without differences between experimental diets. From the analysis of phyla abundances in the current study it is obvious that the taxonomic dominance shifts towards Proteobacteria, Fusobacteria and Actinobacteria in fish that were fed with A and B as first feeding diet. In trout fry, grown on the first feeding diet C, Proteobacteria were already present in a relative abundance of about 50% and thus the richness was already higher in comparison. There are several factors that could have influenced the increase of species: First of all, the diet change could have induced an alteration of the established intestinal environment and thus attracted additional bacteria on top of the ones that colonized the GI tract during the first feeding period. Second however, species richness also increased when no diet change was performed and so intrinsic factors could be emerging during the morphological development of young trout fry, like changes in the pH or the growth of the GI tract and thus increased residence time or simply more space. In a study on the bacterial community turnover within developing zebrafish [67] it was found that time was generally a better predictor for species richness and bacterial turnover than was the intestinal volume, which would go in line with the assumption of ontogenetic factors being involved. The Chao1 richness estimator and the Simpson’s evenness measure follow a very similar pattern, which is not surprising with Firmicutes being present in 85% and 90% of all sequences obtained for diet A and diet B, respectively, after the first feeding period. The Shannon diversity index was significantly affected by the diet-type and no nutritional programming effects of the first feeding diet could be detected. Thus, the large difference between diets with and without animal protein, in combination with a stable richness for all fish fed without animal protein reflects the strong influence of both the second feeding diet and intrinsic factors on the development of specific OTUs, and on the overall diversity in the digestive tract of trout fry.

## Conclusions

The intestinal microbiome is substantially involved in metabolism, and increasing plant protein levels in fish feed are significantly affecting this microbiome. This study has furthermore demonstrated that the intestinal microbiome of Rainbow trout can adapt several times towards a given diet and that the diet-type modulates this adaptation. Thus nutritional programming mechanisms of diets on the bacterial community could not be detected—within the presented experimental setup. Early fry feeding with plant proteins will therefore not positively influence the relation between the intestinal microbiome and plant proteins fed in later life stages. However, early fry feeding with fishmeal (representing the current state of work) will certainly not affect the subsequent replacement with plant proteins as well. The phyla and orders found in this experiment agree very well with bacteria that have been found in previous studies and thus the idea of a trout specific core microbiome on higher taxonomic levels is still valid. However, a comparison of the core microbiome in the present study with data from previous results suggests that on lower taxonomic levels diet has a strong modulating effect. These findings should therefore play a key role in further research about fishmeal substitution by including specific bacterial strains commonly present in the GI tract of trout into digestibility analysis.

## Supporting information

S1 Table Complete list of the core microbiota. (PDF)

S1 Text Extended version of the statistical analysis. (PDF)
