# Supplementary material for: The malleable gut microbiome of juvenile rainbow trout (Oncorhynchus mykiss): Diet-dependent shifts of bacterial community structures
Source: PLoS One. 2017 May 12;12(5):e0177735. doi: 10.1371/journal.pone.0177735 (PMC5428975; doi:10.1371/journal.pone.0177735)
Supplement: S1 Table — (PDF) [file pone.0177735.s001.pdf]

S1 Table: Supporting Information.

Complete list of core microbiota (OTUs present in 80% of samples per experimental group).

First Feeding Period (54 dpff)	
**Experimental group**	**OTU**
**Diet A & Diet B & Diet C**	[u'p__Proteobacteria', u'c__Betaproteobacteria', u'o__Burkholderiales', u'f__Oxalobacteraceae', u'g__', u's__']
	[u'p__Firmicutes', u'c__Bacilli', u'o__Bacillales', u'f__Staphylococcaceae', u'g__Staphylococcus', u's__']
**Diet A & Diet B**	[u'p__Firmicutes', u'c__Bacilli', u'o__Bacillales', u'f__Staphylococcaceae', u'g__Staphylococcus', u's__']
	[u'p__Firmicutes', u'c__Bacilli', u'o__Bacillales', u'f__Staphylococcaceae', u'g__Staphylococcus', u's__']
	[u'p__Firmicutes', u'c__Bacilli', u'o__Bacillales', u'f__Staphylococcaceae', u'g__Staphylococcus', u's__']
**Diet B & Diet C**	[u'p__Proteobacteria', u'c__Betaproteobacteria', u'o__Burkholderiales', u'f__Burkholderiaceae', u'g__Burkholderia', u's__']
**Diet A**	[u'p__Firmicutes', u'c__Bacilli', u'o__Bacillales', u'f__Staphylococcaceae', u'g__Staphylococcus', u's__']
	[u'p__Firmicutes', u'c__Bacilli', u'o__Bacillales', u'f__Staphylococcaceae', u'g__Staphylococcus', u's__aureus']
	[u'p__Firmicutes', u'c__Bacilli', u'o__Lactobacillales', u'f__Streptococcaceae']
	[u'p__Actinobacteria', u'c__Actinobacteria', u'o__Actinomycetales', u'f__Corynebacteriaceae', u'g__Corynebacterium', u's__']
	[u'p__Firmicutes', u'c__Bacilli', u'o__Bacillales', u'f__Staphylococcaceae', u'g__Staphylococcus', u's__']
	[u'p__Proteobacteria', u'c__Alphaproteobacteria', u'o__Rhodobacterales', u'f__Rhodobacteraceae', u'g__Rhodobacter', u's__']
	[u'p__Firmicutes', u'c__Bacilli', u'o__Bacillales', u'f__Staphylococcaceae', u'g__Staphylococcus', u's__']

	[u'p__Firmicutes', u'c__Bacilli', u'o__Bacillales', u'f__Staphylococcaceae', u'g__Staphylococcus', u's__epidermidis']
	[u'p__Firmicutes', u'c__Bacilli', u'o__Bacillales', u'f__Staphylococcaceae', u'g__Staphylococcus', u's__']
**Diet B**	[u'p__Firmicutes', u'c__Bacilli', u'o__Bacillales', u'f__Staphylococcaceae', u'g__Staphylococcus', u's__']
	[u'p__Proteobacteria', u'c__Alphaproteobacteria', u'o__Rhizobiales', u'f__Phyllobacteriaceae']
**Diet C**	[u'p__Firmicutes', u'c__Bacilli', u'o__Lactobacillales', u'f__Lactobacillaceae', u'g__Lactobacillus', u's__']
	[u'p__Firmicutes', u'c__Bacilli', u'o__Bacillales', u'f__Bacillaceae', u'g__Bacillus', u's__']
	[u'p__Bacteroidetes', u'c__Flavobacteriia', u'o__Flavobacteriales', u'f__[Weeksellaceae]', u'g__Cloacibacterium', u's__']
	[u'p__Proteobacteria', u'c__Betaproteobacteria', u'o__Burkholderiales', u'f__Oxalobacteraceae', u'g__Janthinobacterium', u's__lividum']
	[u'p__Proteobacteria', u'c__Alphaproteobacteria', u'o__Rhodospirillales', u'f__Acetobacteraceae', u'g__Acetobacter', u's__']
	[u'p__Proteobacteria', u'c__Gammaproteobacteria', u'o__Pseudomonadales', u'f__Moraxellaceae', u'g__Acinetobacter', u's__rhizosphaerae']
	[u'p__Firmicutes', u'c__Bacilli', u'o__Lactobacillales', u'f__Streptococcaceae', u'g__Lactococcus', u's__']
	[u'p__Firmicutes', u'c__Bacilli', u'o__Lactobacillales', u'f__Streptococcaceae', u'g__Streptococcus', u's__']
	[u'p__Proteobacteria', u'c__Alphaproteobacteria', u'o__Rhodospirillales', u'f__Rhodospirillaceae', u'g__', u's__']
	[u'p__Firmicutes', u'c__Bacilli', u'o__Lactobacillales', u'f__Lactobacillaceae', u'g__Lactobacillus', u's__']
	[u'p__Firmicutes', u'c__Bacilli', u'o__Lactobacillales', u'f__Lactobacillaceae', u'g__Lactobacillus', u's__']
	[u'p__Proteobacteria', u'c__Gammaproteobacteria', u'o__Pseudomonadales', u'f__Moraxellaceae', u'g__Acinetobacter', u's__']
	[u'p__Proteobacteria', u'c__Gammaproteobacteria', u'o__Pseudomonadales', u'f__Moraxellaceae', u'g__Acinetobacter', u's__johnsonii']
	[u'p__Proteobacteria', u'c__Alphaproteobacteria', u'o__Rhodospirillales', u'f__Acetobacteraceae', u'g__Acetobacter', u's__']
	[u'p__Proteobacteria', u'c__Gammaproteobacteria', u'o__Pseudomonadales', u'f__Moraxellaceae', u'g__', u's__']

	[u'p__Proteobacteria', u'c__Betaproteobacteria', u'o__Burkholderiales', u'f__Comamonadaceae', u'g__Variovorax', u's__paradoxus']
	[u'p__Firmicutes', u'c__Bacilli', u'o__Bacillales', u'f__Bacillaceae', u'g__Bacillus', u's__coagulans']
	[u'p__Proteobacteria', u'c__Gammaproteobacteria', u'o__Enterobacteriales', u'f__Enterobacteriaceae', u'g__Klebsiella', u's__']
	[u'p__Firmicutes', u'c__Bacilli', u'o__Lactobacillales', u'f__Leuconostocaceae', u'g__Leuconostoc', u's__']
	[u'p__Proteobacteria', u'c__Gammaproteobacteria', u'o__Enterobacteriales', u'f__Enterobacteriaceae', u'g__Klebsiella', u's__']
	[u'p__Firmicutes', u'c__Bacilli', u'o__Lactobacillales', u'f__Lactobacillaceae', u'g__Lactobacillus', u's__']
	[u'p__Proteobacteria', u'c__Betaproteobacteria', u'o__Burkholderiales', u'f__Oxalobacteraceae', u'g__Janthinobacterium', u's__lividum']
	[u'p__Firmicutes', u'c__Bacilli', u'o__Lactobacillales', u'f__Streptococcaceae', u'g__Streptococcus', u's__']
	[u'p__Firmicutes', u'c__Bacilli', u'o__Lactobacillales', u'f__Streptococcaceae', u'g__Streptococcus', u's__']
**Second Feeding Period (93 dpff)**	
**Diet A & Diet B**	[u'p__Proteobacteria', u'c__Gammaproteobacteria', u'o__Alteromonadales', u'f__Moritellaceae', u'g__Moritella', u's__']
	[u'p__Proteobacteria', u'c__Gammaproteobacteria', u'o__Vibrionales', u'f__Vibrionaceae', u'g__Photobacterium', u's__']
	[u'p__Fusobacteria', u'c__Fusobacteriia', u'o__Fusobacteriales', u'f__Fusobacteriaceae', u'g__Psychrilyobacter', u's__']
	[u'p__Bacteroidetes', u'c__Bacteroidia', u'o__Bacteroidales', u'f__Bacteroidaceae', u'g__Bacteroides', u's__']
	[u'p__Proteobacteria', u'c__Gammaproteobacteria', u'o__Vibrionales', u'f__Vibrionaceae', u'g__Aliivibrio', u's__fischeri']
	[u'p__Firmicutes', u'c__Bacilli', u'o__Lactobacillales', u'f__Streptococcaceae']
	[u'p__Bacteroidetes', u'c__Bacteroidia', u'o__Bacteroidales', u'f__Porphyromonadaceae', u'g__Porphyromonas', u's__']
	[u'p__Bacteroidetes', u'c__Bacteroidia', u'o__Bacteroidales', u'f__Bacteroidaceae', u'g__Bacteroides', u's__']

	[u'p__Proteobacteria', u'c__Gammaproteobacteria', u'o__Alteromonadales', u'f__Moritellaceae', u'g__Moritella', u's__']
	[u'p__Proteobacteria', u'c__Gammaproteobacteria', u'o__Pseudomonadales', u'f__Moraxellaceae', u'g__Acinetobacter', u's__johnsonii']
	[u'p__Bacteroidetes', u'c__Bacteroidia', u'o__Bacteroidales', u'f__Bacteroidaceae', u'g__Bacteroides', u's__']
	[u'p__Fusobacteria', u'c__Fusobacteriia', u'o__Fusobacteriales', u'f__Fusobacteriaceae', u'g__Fusobacterium', u's__']
	[u'p__Fusobacteria', u'c__Fusobacteriia', u'o__Fusobacteriales', u'f__Fusobacteriaceae', u'g__u114', u's__']
	[u'p__Proteobacteria', u'c__Gammaproteobacteria', u'o__Vibrionales', u'f__Pseudoalteromonadaceae', u'g__Pseudoalteromonas', u's__']
	[u'p__Firmicutes', u'c__Bacilli', u'o__Lactobacillales', u'f__Enterococcaceae', u'g__Vagococcus', u's__']
	[u'p__Fusobacteria', u'c__Fusobacteriia', u'o__Fusobacteriales', u'f__Fusobacteriaceae', u'g__Fusobacterium', u's__']
	[u'p__Proteobacteria', u'c__Gammaproteobacteria', u'o__Alteromonadales', u'f__Shewanellaceae']
**Diet B & Diet C**	[u'p__Proteobacteria', u'c__Gammaproteobacteria', u'o__Pseudomonadales', u'f__Moraxellaceae', u'g__Acinetobacter', u's__rhizosphaerae']
	[u'p__Proteobacteria', u'c__Betaproteobacteria', u'o__Burkholderiales', u'f__Oxalobacteraceae', u'g__', u's__']
	[u'p__Firmicutes', u'c__Bacilli', u'o__Lactobacillales', u'f__Streptococcaceae', u'g__Lactococcus', u's__']
	[u'p__Firmicutes', u'c__Bacilli', u'o__Lactobacillales', u'f__Streptococcaceae', u'g__Streptococcus', u's__']
	[u'p__Firmicutes', u'c__Bacilli', u'o__Lactobacillales', u'f__Lactobacillaceae', u'g__Lactobacillus', u's__']
	[u'p__Proteobacteria', u'c__Gammaproteobacteria', u'o__Pseudomonadales', u'f__Moraxellaceae', u'g__Acinetobacter', u's__']
	[u'p__Firmicutes', u'c__Bacilli', u'o__Bacillales', u'f__Staphylococcaceae', u'g__Staphylococcus', u's__']
	[u'p__Proteobacteria', u'c__Gammaproteobacteria', u'o__Pseudomonadales', u'f__Moraxellaceae', u'g__', u's__']
	[u'p__Firmicutes', u'c__Bacilli', u'o__Lactobacillales', u'f__Streptococcaceae', u'g__Streptococcus', u's__']
	[u'p__Firmicutes', u'c__Bacilli', u'o__Lactobacillales', u'f__Streptococcaceae', u'g__Streptococcus', u's__']

**Diet A**	[u'p__Bacteroidetes', u'c__Bacteroidia', u'o__Bacteroidales', u'f__Bacteroidaceae', u'g__Bacteroides', u's__']
	[u'p__Firmicutes', u'c__Clostridia', u'o__Clostridiales', u'f__[Tissierellaceae]', u'g__', u's__']
	[u'p__Fusobacteria', u'c__Fusobacteriia', u'o__Fusobacteriales', u'f__Fusobacteriaceae', u'g__Cetobacterium', u's__somerae']
	[u'p__Proteobacteria', u'c__Gammaproteobacteria', u'o__Aeromonadales', u'f__Aeromonadaceae', u'g__', u's__']
	[u'p__Fusobacteria', u'c__Fusobacteriia', u'o__Fusobacteriales', u'f__Fusobacteriaceae', u'g__Psychrilyobacter', u's__']
	[u'p__Bacteroidetes', u'c__Flavobacteriia', u'o__Flavobacteriales', u'f__Flavobacteriaceae', u'g__Myroides', u's__odoratimimus']
	[u'p__Firmicutes', u'c__Clostridia', u'o__Clostridiales', u'f__[Mogibacteriaceae]', u'g__', u's__']
	[u'p__Bacteroidetes', u'c__Bacteroidia', u'o__Bacteroidales', u'f__Bacteroidaceae', u'g__Bacteroides', u's__']
	[u'p__Fusobacteria', u'c__Fusobacteriia', u'o__Fusobacteriales', u'f__Fusobacteriaceae', u'g__Fusobacterium', u's__']
	[u'p__Firmicutes', u'c__Bacilli', u'o__Lactobacillales', u'f__Streptococcaceae', u'g__Streptococcus', u's__']
	[u'p__Bacteroidetes', u'c__Bacteroidia', u'o__Bacteroidales', u'f__Bacteroidaceae', u'g__Bacteroides']
**Diet B**	[u'p__Firmicutes', u'c__Bacilli', u'o__Lactobacillales', u'f__Leuconostocaceae']
	[u'p__Proteobacteria', u'c__Gammaproteobacteria', u'o__Enterobacteriales', u'f__Enterobacteriaceae', u'g__Klebsiella', u's__']
	[u'p__Firmicutes', u'c__Bacilli', u'o__Lactobacillales', u'f__Lactobacillaceae', u'g__Lactobacillus', u's__']
**Diet C**	[u'p__Firmicutes', u'c__Bacilli', u'o__Lactobacillales', u'f__Lactobacillaceae', u'g__Lactobacillus', u's__']
	[u'p__Proteobacteria', u'c__Gammaproteobacteria', u'o__Pseudomonadales', u'f__Moraxellaceae', u'g__Acinetobacter', u's__']
	[u'p__Firmicutes', u'c__Bacilli', u'o__Lactobacillales', u'f__Leuconostocaceae', u'g__Leuconostoc', u's__']
