# Supplementary material for: The malleable gut microbiome of juvenile rainbow trout (Oncorhynchus mykiss): Diet-dependent shifts of bacterial community structures
Source: PLoS One. 2017 May 12;12(5):e0177735. doi: 10.1371/journal.pone.0177735 (PMC5428975; doi:10.1371/journal.pone.0177735)
Supplement: S1 Text — (PDF) [file pone.0177735.s002.pdf]

S1 Text: Supporting Information

Extended version of the statistical analysis

The statistical software R [1] and QIIME were used to evaluate the data, generated on days 54 and 93 post first feeding. Important to note here is the small number of samples obtained for diet C at sampling day 54 post first feeding: from five GI-tract samples extracted, only three samples showed substantial sequencing results and could thus be integrated into the statistical analysis. Four alpha diversity measures were calculated based on the complete OTU table generated during the QIIME workflow. The number of observed distinct OTUs, the good's coverage estimator, the Chao1 richness estimator, the Simpson's evenness measure E and the Shannon diversity index H' were selected for the purpose of estimating alpha diversity of microbial species within the GI tract samples. Differences between bacterial communities in relation to the dietary treatment or sampling day were visualized by nonmetric multidimensional scaling (NMDS) based on a Bray-Curtis dissimilarity matrix of Hellinger-transformed abundance data on order level using the R package vegan [2]. A stress factor was calculated to provide a way of determining how well original data is represented in the ordination space. The core microbiota of fish fed the different experimental diets at the end of the first feeding period and at the end of the second feeding period was calculated with QIIME by using the complete OTU table generated during bioinformatics workflow. The core microbiota of the present study was defined as OTUs present in 80% of samples from each dietary treatment. By using an open access web tool, Venn diagrams were drawn to visualize the core microbiota (http://bioinformatics.psb.ugent.be/webtools/Venn/). The core microbiota at the end of the second feeding period was calculated for the three second feeding diets only and all samples were pooled for their respective first feeding diet.

Further statistical analysis of the data was performed as follows:

First, the influence of the three first feeding diets (A, B and C) on the four alpha diversity indices and on the top-five most abundant bacterial phyla at the end of the first feeding period was tested. Therefore a statistical model based on generalized least squares was established [3]. The data were assumed to be approximately normally distributed and to be heteroscedastic. These assumptions were based on a graphical residual analysis. The first feeding diet was considered as (fixed) factor. Based on this model, an analysis of variances (ANOVA) was conducted and multiple contrast tests were performed [4] using the R package SimComp [5] to compare the three different first feeding diets.

Second, the influence of the nine dietary treatments on alpha diversity indices and on the top-five most abundant phyla at the end of the second feeding period was tested. Thus, a statistical mixed model was defined [6, 7] with the first feeding diet and the second feeding diet as well as their interaction term as fixed factors. Again, the data were assumed to be approximately normally distributed and heteroscedastic. The individual aquaria were included as random factor. Based on this model, an ANOVA was conducted followed by multiple contrast tests to compare the levels of the fixed factors [8, 9] using the R package multcomp [10]. A significant interaction of the first feeding diet and the second feeding diet was considered as nutritional programming effect of the first feeding diet. In case of a non-significant interaction, data were pooled for the first feeding diet and multiple contrast tests were rerun to compare only the three second feeding diets (A, B and C).

Third, statistical differences of alpha diversity indices and bacterial phyla between the end of the first feeding period (day 54 pff) and the end of the second feeding period (day 93 pff) were evaluated for continuously fed fish (treatments A, AA, B, BB, C and CC). A statistical mixed model was established with the sampling day as fixed factor and the hatching troughs and aquaria as random factor. An ANOVA was conducted, followed by multiple contrast tests to compare the two sampling days as described before [8, 10].

Fourth, the influence of the three first feeding diets on relative bacterial abundances at the end of the first feeding period was tested. Abundance data were evaluated on bacterial order level. This was a necessary compromise between a precise (sequencing depth) and a robust statistical analysis. Data were Hellinger-transformed and a Principal Component Analysis (PCA) was performed [11]. The Broken-Stick-Criterion [12] was used to select those principal components (PC) from the PCA with the greatest influence on data variability. The first two PC's represented 85% of the cumulative variance. Based on these two PC's, rotated data (i.e. two pseudo-variables) were calculated and integrated into a multivariate model, established simultaneously for the two pseudo-variables. An ANOVA was performed based on this model and multiple contrast tests were conducted in order to compare the three diets simultaneously for the two pseudo-variables [5, 13].

Fifth, the influence of the nine dietary treatments on the bacterial community structure at the end of the second feeding period was tested. A PCA was performed with Hellinger-transformed abundance data on order level and the PC's with the highest influence on data variability were selected as described before. The first six PC's represented 84% of the cumulative variance. Based on these six PC's, rotated data were calculated and integrated into a multivariate mixed model, established simultaneously for the six pseudo-variables. The first feeding diet and the second feeding diet as well as their interaction were considered as fixed factors, the aquaria were considered as random factor. Based on this model, an ANOVA was conducted. Again, a significant interaction of the first feeding diet and the second feeding diet was considered as nutritional programming effect of the first feeding diet. Afterwards, multiple contrast tests were performed to compare the levels of the fixed factors simultaneously for the six pseudo-variables [5, 13].

Sixth, the first two PC's were further examined for the individual contribution of specific bacterial orders to the cumulative variance explained of each principal component. The top-ten orders with the highest loadings on each of the two PC's were selected for further analysis. In case of a non-significant interaction of the first and the second feeding diet in the

previous model, data were pooled for the first feeding diet and multiple contrast tests as described before [5, 13] were performed to compare the three second feeding diets (A, B and C) simultaneously for the ten selected bacterial orders, respectively for each PC. Thus, specific bacterial orders could be identified that were significantly promoted by a certain diet-type.

Seventh, statistical differences of the bacterial community structure between the end of the first feeding period (day 54 pff) and the end of the second feeding period (day 93 pff) were evaluated for continuously fed fish (treatments A and AA, B and BB, C and CC, respectively). A PCA was performed for each of the three Hellinger-transformed data pairs and the first three PC's were selected as described before. Resulting pseudo-variables were integrated into a multivariate mixed model established simultaneously for the three pseudo-variables. The sampling day was integrated as fixed factor and the hatching troughs and aquaria as random factor. An ANOVA was conducted, followed by multiple contrast tests to compare the two sampling days simultaneously for the three pseudo-variables as described before [5, 13].

Finally, a correlation analysis based on Spearman ranks was conducted in order to evaluate a possible relation between the bodymass of individual fish and the first two principal components of the PCA. The correlation analysis was repeated for each of the second feeding diets. For PC2 a significant correlation was found and the top ten orders with the highest loadings on this PC were used again in a Spearman ranks correlation analysis to test possible relations of a specific bacterial order to bodymass.

References

1. R Core Team. R: A language and environment for statistical computing. Vienna, Austria: R Foundation for Statistical Computing; 2006.

2. Oksanen J, Blanchet FG, Kindt R, Legendre P, Minchin PR, O'Hara RB, et al. vegan: Community Ecology Package; 2016.

3. Carroll RJ, Ruppert D. Transformation and weighting in regression. 1st ed. New York NY u.a: Chapman & Hall; 1988.

4. Hasler M, Hothorn LA. Multiple contrast tests in the presence of heteroscedasticity. Biom J. 2008;50:793–800. doi:10.1002/bimj.200710466.

5. Hasler M. SimComp: Simultaneous Comparisons for Multiple Endpoints; 2014.

6. Verbeke G, Molenberghs G. Linear mixed models for longitudinal data: Springer; 2000.

7. Laird NM, Ware JH. Random-Effects Models for Longitudinal Data. Biometrics. 1982;38:963. doi:10.2307/2529876.

8. Bretz F, Hothorn T, Westfall PH. Multiple comparisons using R. Boca Raton, FL: Chapman and Hall / CRC Press; 2011.

9. Schaarschmidt F, Vaas L. Analysis of trials with complex treatment structure using multiple contrast tests. HortScience. 2009;44:188–95.

10. Hothorn T, Bretz F, Westfall P, Heiberger RM. Multcomp: Simultaneous Inference in General Parametric Models—R Package Version 1.0–0. R Foundation for Statistical Computing. Vienna, Austria. 2008.

11. Hartung J, Elpelt B. Multivariate Statistik: Lehr- und Handbuch der angewandten Statistik. 6th ed. München: Oldenbourg; 1999.

12. Jackson DA. Stopping rules in principal components analysis: a comparison of heuristical and statistical approaches. Ecology. 1993;74:2204–14.

13. Hasler M. Multiple contrast tests for multiple endpoints in the presence of heteroscedasticity. Int J Biostat. 2014;10:17–28. doi:10.1515/ijb-2012-0015.
